# EMPOWERING BEHAVIOR CHANGE TO SUPPORT BRAIN HEALTH AMONG OLDER ADULTS

**DOI:** 10.1093/geroni/igac059.211

**Published:** 2022-12-20

**Authors:** Sarah Lock, Duke Han

## Abstract

This symposium will provide highlights from the Global Council on Brain Health’s (GCBH) body of work on behavior change. This work is focused on developing a better understanding of (a) how to persuade and motivate people to engage in sustained healthy behaviors, (b) how to change policies within local communities to support individuals’ ability to make healthy choices, and (c) how to optimize conditions for brain health so all can thrive. The GCBH is an independent collaborative of scientists, clinicians, scholars, and policy experts convened by AARP to provide evidence-based advice on what people and professionals can do to maintain and improve brain health. The Council translates scientific research into actionable recommendations aimed at helping to drive behavior change in individuals across communities and cultures. Experts were brought together to build consensus around a range of factors at the individual and societal level that influence individual behavior and decision-making. This presentation will also draw upon lessons learned from the field of health promotion and examples from communication campaigns around heart health will be discussed as they relate to brain health. Nationally representative surveys, fielded by AARP Research including diverse respondents of adults 40+ and health care professionals diagnosing and treating dementia, found that misperceptions and stigma about dementia are commonplace and hinder efforts to address brain-healthy behaviors. Physicians who used to treat dementia patients, now themselves living with dementia, discuss how championing change, providing hope and refuting stigma can even help those living with a dementia diagnosis.

